# Effects of Cognitive Behavioral Therapy–Based Intervention on Improving Glycaemic, Psychological, and Physiological Outcomes in Adult Patients With Diabetes Mellitus: A Meta-Analysis of Randomized Controlled Trials

**DOI:** 10.3389/fpsyt.2020.00711

**Published:** 2020-07-28

**Authors:** Xiangyun Yang, Zhanjiang Li, Jing Sun

**Affiliations:** ^1^ The National Clinical Research Center for Mental Disorders & Beijing Key Laboratory of Mental Disorders, Beijing Anding Hospital, Capital Medical University, Beijing, China; ^2^ Advanced Innovation Center for Human Brain Protection, Capital Medical University, Beijing, China; ^3^ School of Medicine, Griffith University, Gold Coast, QLD, Australia

**Keywords:** cognitive behavioral therapy, diabetes mellitus, glycaemic control, mood symptoms, meta-analysis

## Abstract

**Background:**

Patients with diabetes mellitus (DM) have a high risk of secondary physiological and psychological complications. Some interventions based on cognitive behavioral therapy (CBT) have been used to control glucose levels and improve negative emotions of patients with DM. This study was undertaken to provide an overview of the effectiveness of CBT-based interventions for improving glycaemic control, psychological, and physiological outcomes in adult patients with DM.

**Methods:**

Randomized controlled trials (RCTs) published in English and Chinese during 2007 and April 2019 were searched through various electronic databases including PubMed, Cochrane Library, Scopus, Embase, ProQuest Dissertations and Theses, and the Chinese databases (WanFang data and China National Knowledge Infrastructure). The primary outcome variables included glycated haemoglobin (HbA1c), fasting plasma glucose (FPG), depression, and anxiety symptoms. The secondary outcomes were weight and cholesterol. Effect sizes were pooled by random-effects modelling using Comprehensive Meta-Analysis software. Physiotherapy Evidence Database tool was used to assess the quality of all included studies.

**Results:**

Twenty-three RCTs comprising 2,619 patients with DM (type 1 and type 2) were included in at least one meta-analysis. The results of the main analysis showed that CBT-based interventions had a better effect on reduced HbA1c (−0.275%, 95% CI: −0.443 to −0.107; p < 0.01) with Hedge’s g of 0.466 (95% CI: 0.710 – 0.189), reduced depression symptoms with average reduction of −2.788 (95% CI: −4.450 to −1.207; p < 0.01) and Hedge’s g of 0.966 (95% CI: 1.507 – 0.426). Twenty-three RCTs comprising 2,619 patients with DM (type 1 and type 2) were included in this meta-analysis. Several mediators of the effect were found through subgroup analysis for HbA1c and depression symptoms. The interventions emphasising completion homework assignments, stress management, and that used an interpersonal strategy delivered *via* a group had a better effect on both HbA1c and depression symptoms. In addition, behavioral strategies had a better effect on glycaemic control, and cognitive strategies had a better effect on depression symptoms. There was no difference in the change of FPG, anxiety symptoms, weight, and high-density lipoprotein cholesterol (HDL-C) between CBT-based interventions and the control conditions.

**Conclusions:**

The findings indicate that CBT-based interventions are effective for improving glycaemic control and depression symptoms in adult patients with type 1 DM (T1DM) or type 2 DM (T2DM) with moderate to large effect size. The results of the subgroup analysis suggest that it is necessary to adopt different types and technical components of CBT according to the population and purpose of the treatment in clinical practice. Due to the high heterogeneity of included studies and other limitations, further study including large number of studies is needed to confirm these results.

## Introduction

Diabetes mellitus (DM) is a chronic progressive disease with a high risk of comorbid physical and mental health problems (1). Lower glycated haemoglobin (HbA1c) is helpful in reducing the risk of developing DM-related complications. However, more than 60% of patients with DM have persistent worse glycaemic control (2, 3). The inadequate self-care, non-adherence to medications and combined emotional problems may be the main causes of suboptimal glycaemic control. Apart from physical comorbidity, depression and anxiety symptoms are also common complications in patients with DM. Meanwhile, the mood symptoms may create further obstacles to self-care and treatment adherence, and result in worse glycaemic control and quality of life (4). Thus, the improvement of both glycaemic control and mood symptoms is important in the management of DM.

The American Diabetes Association (ADA) states that patients with DM need comprehensive medical care. In addition to standard drug treatment, reasonable diet, exercise, self-management and psychological intervention are also important in improving any therapeutic effect (1). Recent reviews suggest that psychological intervention can help to improve DM-related outcomes. Chapman and colleagues performed a meta-analysis exploring the effect of psychological interventions—cognitive behavioral therapy (CBT), motivational interviewing (MI) and client-centred therapy—in treating patients with type 2 DM (T2DM) in China. They found CBT and MI were more effective than the control conditions in the reduction of HbA1c. CBT and client-centered therapy were also associated with reductions in depression and blood glucose concentration, and CBT was associated with reductions in anxiety. The results suggest that CBT has positive effect on both glycaemic control and mood management (5). However, another meta-analysis on the effect of psychological interventions (counselling, CBT, family systems therapy, and psychodynamic therapy) on children and adults with type 1 DM (T1DM) suggested that psychological treatments were helpful in improving glycaemic control in children and adolescents, but not in adults (6). Recently, Winkley and colleagues further examined the effectiveness of psychological interventions for glycaemic control in children and adults with TIDM. The aggregate meta-analysis did not find reduction in HbA1c for children or adults. While, the network meta-analysis for adults showed that CBT-based interventions have potential positive effects on HbA1c reduction (7).

Overall, CBT is the most commonly applied and investigated type of psychotherapy for the management of DM. As a structured short-term psychotherapy, CBT has been found to be an effective way of treating a variety of mental disorders, particularly depression (8). This therapy helps individuals to reorganize dysfunctional thoughts, beliefs, and negative behaviors, and then reconstruct appropriate thinking patterns and behavior, which results in better mood adjustment. Currently, CBT has been used to improve the management of chronic physical diseases, including DM (9). Researchers have compiled a specific CBT manual for DM, which has a specific plan for each session and encourages the individual to actively practice through homework assignments (10). It is well-accepted that thoughts, beliefs, behaviors, feelings and physiology are integrated and affect the way the patient manages their diabetes. CBT-based interventions could enhance patients’ awareness about the relationship between glucose control and negative thoughts, behaviors and feelings in relation to DM. It may also help patients to create better self-care behavior and achieve better glycaemic control than simply just practice exercise and diet control regime.

One early review and meta-analysis (including four studies) specifically examined the effect of CBT for glycaemic control, and did not find overall statistically significant effect (11). Uchendu and colleagues performed a review and meta-analysis on the effectiveness of CBT for patients with both T1DM and T2DM. They analyzed nine randomized controlled trials (RCTs) using single CBT as the intervention and found that CBT had short-term and medium-term benefits in improving glycaemic control, and short-term, medium-term, and long-term benefits for depression (12). Another meta-analysis reviewed five studies on the effect of CBT on depression symptoms in patients with T1DM or T2DM, and demonstrated that CBT can improve depressive mood effectively regardless of the type of diabetes (13). Li and colleagues conducted a review and meta-analysis including 10 RCTs to explore the effect of CBT for diabetic patients comorbid with depression and found CBT was effective in reducing depression symptoms, fasting glucose and improving quality of life and anxiety in patients with diabetes in comorbid with depression (14).

The above reviews and meta-analyses have found trend for the beneficial effect of CBT in improving glycaemic control and mood management of patients with DM. However, these findings could not fully demonstrate the effect of full spectrum of CBT-based intervention components in patients with DM. This is because CBT has experienced a rapid development of the “third wave” during the past years and several new branches of CBT have emerged in recent years. These new components include mindfulness-based cognitive therapy (MBCT), acceptance and commitment therapy (ACT), metacognitive therapy, dialectical behavioral therapy, and schema therapy (15, 16). Recent studies have used MBCT and ACT to treat patients with DM since 2007 (17–19) and some studies have developed CBT-based intervention through web and mobile phone applications in the treatment of DM (20–22). All of these newly developed CBT-based interventions may potentially improve glycaemic control and psychological disorders in patients with DM. Moreover, many studies have applied CBT in combination with other methods, including motivational interviewing, physical activity and education, to improve the management of DM (23–25). However, these newly developed CBT-based interventions have not been reviewed and synthesized by a meta-analysis. This study aimed to fill in this research gap to perform an expanded meta-analysis to evaluate whether comprehensive CBT-based interventions have a positive effect on glycaemic control, mood symptoms, and other physical outcomes in adult patients with either T1DM or T2DM. Thus, current meta-analyses aimed to examine RCTs using CBT alone or combined CBT, traditional CBT or newly developed CBT (MBCT and ACT), and CBT that is delivered face-to-face or remotely to expand the understanding of the effectiveness of CBT-based interventions for patients with DM.

## Methods

### Literature Search Strategy

The protocol of this review was registered at the Prospero International Prospective Register of Systematic Reviews (Registration ID: CRD42019132012 PROSPERO 2019; website: https://www.crd.york.ac.uk/prospero/display_record.php?RecordID=132012).

Two reviewers searched all relevant literature using the following electronic databases: PubMed, Cochrane Library, Scopus, Embase, ProQuest Dissertations and Theses, and the Chinese databases including WanFang data and Chinese National Knowledge Infrastructure (CNKI). The studies were restricted to peer-reviewed journal articles for the period 2007 to April 2019 because the newly CBT-based intervention (MBCT and ACT) was used for the management of diabetic patients since 2007. The following keywords were used in the search process: “diabetes mellitus”, “type 1 diabetes”, “type 2 diabetes”, “cognitive behavioral (behavioral) therapy”, “cognitive therapy”, “behavioral (behavioral) therapy” “mindfulness-based cognitive therapy”, “accept and commitment”, and “randomized/randomised controlled”, with Boolean operators AND and OR, and with language limitation on English and Chinese. In the process of retrieval, the search terms were modified according to the search rules for the different databases (**Supplementary Table 1**). We also searched the reference lists of the original papers to find additional relevant articles.

### Inclusion and Exclusion Criteria

The inclusion criteria of the studies in this meta-analysis were as follows:

1. The study had male and female participants aged 18 years or older.
2. The participants were non-hospitalized patients diagnosed with either T1DM or T2DM.
3. The study design was an RCT (pragmatic controlled trial was also eligible). The intervention was mainly based on CBT strategies (the strategies must be under the umbrella of CBT included cognitive therapy, behavioral therapy, ACT, or MBCT), including common CBT techniques such as relaxation, goal-setting, behavioral experiment, cognitive re-structuring. The interventions could be CBT alone or CBT combined with other methods, delivered face-to-face or remotely (e.g., *via* telephone and internet) and used an individual form or a group form. The control conditions included non-CBT interventions (e.g., medication, education), or usual care or waiting list.
4. The primary outcome variables were glycaemic control (HbA1c and fasting blood glucose) and mood symptoms (depression symptoms and anxiety symptoms). The secondary outcomes included total cholesterol, HDL-C, weight. In addition, all indexes had to have been measured using validated methods.

The exclusion criteria of the studies in this meta-analysis were as follows:

1. The patients had gestational DM or were pre-diabetes.
2. There were no available data.
3. The patients were hospitalized.
4. If there were duplicate publications for the same sample. In such cases, only the most recent study was included in our analysis.
5. The studies were of low quality, with a Physiotherapy Evidence Database tool (PEDro) of ≤3.
6. The language was not English or Chinese.
7. Non peer-reviewed journal articles.

### Paper Screening and Data Extraction

Two authors screened all the articles according to the Preferred Reporting Items for Systematic Reviews and Meta-analysis (PRISMA) guidelines. After discarding duplicate studies, two authors independently assessed all paper’s eligibility by reading the title and abstract. Any discrepancies were resolved through discussion with a third author. Then, they read the full text of eligible papers to determine which studies to include.

A data-collection form was then used to extract data on the characteristics of the study and its participants (i.e., study design, study population, sample size, treatment form, frequency and duration of CBT, delivery mode, condition of control group), and on the outcomes measured from the selected studies. The primary outcomes were glycaemic control and severity of mood symptoms. The secondary outcomes were the total cholesterol, weight, and other physical indexes. The data of the Chinese studies were translated into English by two authors (XY and JS) who are fluent in English and Mandarin. For studies with follow-up, we extracted the post-intervention outcomes rather than follow-up outcomes. For the two studies that had incomplete data, we attempted to contact the author, but did not receive a timely response. We therefore extracted only the data reported in the original paper (26, 27).

### Quality Assessment

Two reviewers independently read the full texts of included articles and assessed their methodological quality using the PEDro (28). PEDro includes ten items: random allocation of subjects into groups, concealed randomization, similarity of baseline information between groups, blinding to subjects, blinding to assessors and researchers, attrition rate, use of “intention to treat”, use of variability measures, and use of between-group comparison methods. Based on these ten items, PEDro categorizes the quality of studies into three levels: high quality (8 or more points), moderate quality (4–7 points), and lower quality (3 points or less). There was no obvious disagreement between the two reviewers in rating the quality of the included studies.

### Statistical Analysis

The data of the included trials were analysed using Comprehensive Meta-Analysis software, Version 3.0 (http://www.meta-analysis.com). A separate meta-analysis was performed for each outcome of interest. These included outcomes were reported in at least three studies. The data for variables were converted to the same unit if they were reported with different units in the studies. The pooled mean difference, with a 95% confidence interval (CI) was used for continuous outcome variables, including HbA1c, fasting plasma glucose (FPG), weight, total cholesterol and high-density lipoprotein cholesterol (HDL-C). Standardized mean difference (SMD) and 95% CI were used to measure the severity of depression and anxiety symptoms because the outcomes of the reviewed studies were measured using different instruments. The threshold was set as 0.05 (two sided).

*I^2^* statistic was used to examine the sample heterogeneity between studies. *I^2^* > 50% or *p* < 0.05 indicated the significant heterogeneity. Random effect was performed to calculate the overall mean differences.

Subgroup analyses were conducted for HbA1c and depression symptoms to explore potential factors contributing to heterogeneity and to better understand which types of CBT are more effective. The pooled mean difference was evaluated for each subgroup, and differences in mean differences between the subgroups (with a minimum of three studies) were examined using the Q statistics. The subgroups were the treatment form (individual vs. group); mode of delivery (face-to-face vs. remote); use of a diabetes-specific manual (yes vs. no); number of sessions (<10 vs. ≥10); duration of session (<90 min vs. ≥90min); type of DM (T1DM, T2DM, or both); drop-out rate (<20% vs. ≥20%); intervention type (CBT alone vs. CBT combined with other interventions); patients accompanied by mood symptoms (no vs. yes); and treatment used specific components of CBT (no vs. yes). The definition of using components for CBT was based on the Comprehensive Psychotherapeutic Intervention Rating Scale and previous studies (29–31). The following components of CBT were included in the subgroup analyses: cognitive strategy, behavioral experiment, mood management, stress management, homework assignment, and interpersonal strategies. These components were identified as “yes” (mentioned as an important technique), or “no” (not mentioned and not a core technique).

Publication bias was evaluated using funnel plots and Egger test. A *p*-value of less than 0.05 represented statistically significant publication bias.

## Results

### Search Results

**Figure 1:**
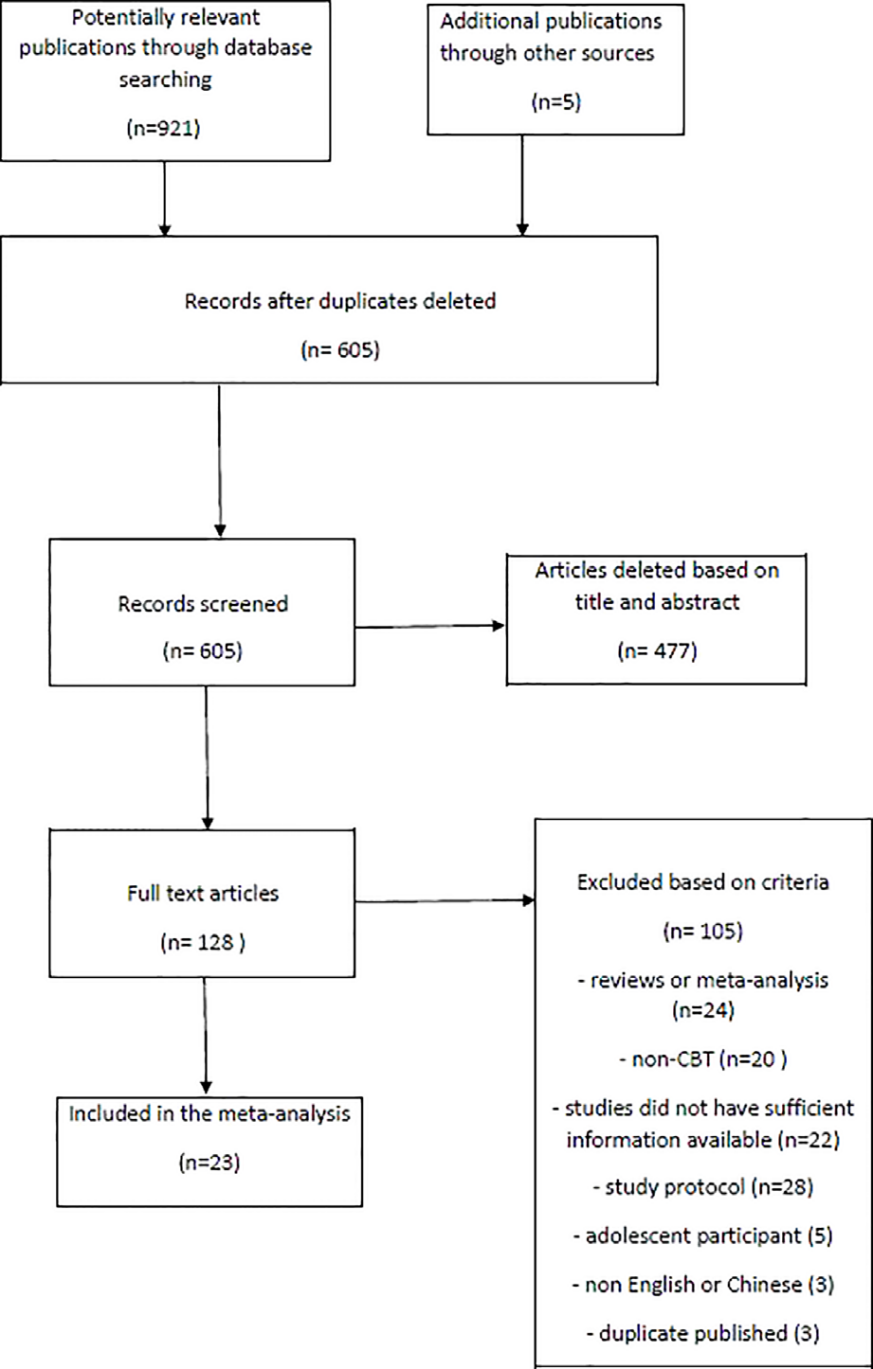
PRISMA flow diagram.

As shown in the PRISMA flow chart (**Figure 1**), 926 articles were identified through the initial search by two assessors. All articles were imported into Endnote, and 321 duplicate articles were removed. The remaining 605 studies were screened for relevance through title and abstract, and 477 articles were then deleted because they did not fulfill the inclusion criteria. Next, 128 full texts were examined carefully according to the inclusion and exclusion criteria of this meta-analysis. Finally, 23 RCTs were included in this systematic review and meta-analysis.

### Characteristics of Included Studies

Full details of the included studies are displayed in **Table 1**. A total of 2,705 community participants were included in the 23 RCTs. These studies were performed in primary, secondary or tertiary care settings in the United States (36, 39, 41, 43, 49), United Kingdom (42, 51), Canada (17), Germany (40), Australia (26), Netherlands (18, 38, 46, 48, 50), Sweden (32), China (34, 37, 47), Iran (44, 45), Saudi Arabia (22), Belgium (33), and Taiwan (35). Of these 23 studies, seven were designed for patients with T1DM, 12 for patients with T2DM, and four for both T1DM and T2DM. Twelve studies had participants that had DM combined with depression and/or anxiety symptoms. Twelve trials adopted CBT alone as an intervention, 11 trials utilized CBT combined with other treatments, including motivational interviewing, education, walking and usual care. CBT-based intervention was delivered face-to face in 19 studies, *via* telephone in two studies, and *via* internet in two studies. Most studies performed traditional CBT, three studies performed ACT, and one study performed MBCT. Most studies conducted a group CBT-based intervention, and four studies conducted individual CBT-based intervention. Eleven studies used usual care as control, 2 studies used waiting-list and 10 studies used non-CBT intervention (education, medication and non-CBT psychotherapy). Other detailed information on the study settings and characteristics of the CBT-based interventions are presented in **Supplementary Table 2**.

**Table 1:** Characteristics of randomized controlled trials included in this meta-analysis.

Study (year)	Participants & ethnicity (No. of C/I baseline(% DO)			Design & Location	Age (C/I)Mean (SD)	Sex M/F (C) M/F (I)	Duration of treatment	Intervention aim		Methods & delivery C/I	KeyIntervention Outcomes(within groups)	Quality of studies assessed by PEDro tool
Alanzi et al. (24)	T2DM patients Saudi Arabia (10/10) (0%/10%)			UB, Saudi Arabia	C, 52.6 (12.6) I, 50.3 (10.5)	C, 7/3 I, 8/2	6 months	Evaluate the effectiveness of SANAD (CBT-based) program in the glycaemic control, health awareness, and self-efficacy on T2DM.		C, usual care I, CBT (SANAD system including using smartphone, social networking services)	-0.60 mean change in HbA1c. 2.44 mean change in diabetes knowledge score 0.994 mean change in self-efficacy score	5, Moderate
Amsberg et al. (32)	T1DM patients Swedish (39/40) (2.7%/10%)			UB, Sweden	C, 41.4 (12.9) I, 41.1 (11.7)	C, 16/22; I, 20/16	48 weeks	Assess CBT-based intervention on HbA1c, self-care behaviors and psychosocial factors among T1DM.		C, usual care (including continuous glucose monitoring) I, same as control plus a CBT-based intervention	-0.78 mean change on HbA1c (48 weeks) -1.63 mean change in anxiety -0.99 mean change in depression	7, Moderate
De Greef et al. (33)	T2DM patients Belgian (21/20) (19%/20%)			UB, Belgium	C, 62.29 (35–75 range) I, 61.5 (35–75 range)	C, 15/6 I, 13/7	12 weeks	Evaluate the benefits of a pedometer and CBT group intervention for physical activity and health in patient with T2DM		C, usual care (including single education session) I, group CBT focus on lifestyle intervention	-0.2 mean change in HbA1c -2.2 mean change in SBP -3.5 mean change in DBP -1.7 mean change in TC 0.4 mean change in Weight -0.1 mean change in BMI	6, Moderate
Guan et al. (34)	T2DM patients Chinese (45/48 (0%/0%)			UB, China	C, 53.6 (7.8) I, 54.4 (8.3)	C, 22/23 I, 25/23	6 months	Effect of group CBT on psychological symptom and glycaemic outcomes in T2DM		C, usual care (including education) I, same as control group plus group CBT	-0.24 mean change in HbA1c -0.39 mean change in fasting glucose	6, Moderate
Gregg et al. (17)	T2DM patients English-speaking Multiple race (38/43) (13%/7%)			UB, Canada	C, 49.8 NR I, 51.9 NR	C, 16/22 I, 22/21	3 months	Assess the effect of ACT and education on glucose control and acceptance coping and self-management inT2DM		C, usual care (education) I, education and ACT (CBT approach based)	-0.72 mean change in HbA1c 5.83 mean change in self-management	8, High
Huang et al. (35)		T2DM patients with depression Taiwanese (30/31) (0%/0%)		UB, Taiwan	C, 57.83 (10.38) I, 55.06 (10.44)	C, 13/17 I, 16/15	3 months		Evaluate effect of motivational enhancement plus CBT on depressive symptom and glucose control of T2DM	C, usual care I, same as control group plus CBT	-3.21 mean change in HbA1c. -2.35 mean change in fasting glucose -1.21 mean change in BMI (kg/m^2^) -5.07 mean change in depression	6, Moderate
Inouye et al. (36)		T2DM patients *Asians and pacific islanders* (103/104) (6.8%/17.3%)		SB, United States	C, 57.8 (10.8) I, 57.0 (11.1)	C, 54/49 I, 59/45	6 weeks		Assess the effect of CBT on quality of life, depression, and glycemia in T2DM	C, usual care (diabetes education) I, CBT	-0.29 mean change in HbA1c 0.4 mean change in BMI 5.3 mean change in SBP 0.1 mean change in DBP -5.5 mean change in TC –2.4 mean change in HDL-C -6.7 mean change in LDL-C -16.4 mean change in Triglyceride 4.5 mean change in weight -2.45 mean change in Depression	9, Moderate
Li et al. (37)		T2DM patients Chinese (20/20) (10%/15%)	UB, China		C, 57.5 (11.32) I: 60.9 (7.87)	C, 10/10 I, 11/9	6 weeks		Evaluate group CBT for emotion and glucose in T2D with	C, usual care (including education) I, same as control group plus group CBT	-0.46 mean change in HbA1c -0.24 mean change in fasting glucose -2.7 mean change in 2hPG	6, Moderate
Menting et al. (38)		T1DM patients *Dutch* (60/60) (0%/1.7%)	UB, Netherlands		C, 42.9 (12.5) I, 44.4 (12.1)	C, 23/37 I, 23/37	5 months		Effectiveness of CBT in reducing fatigue and glucose of T1DM	C, usual care-based waiting list I, CBT (consisting of face-to face and web-based sessions)	-0.1 mean in HbA1c -0.4 mean change in glucose variability -19.4 mean change in Fatigue severity	7, High
Penckofer et al. (39)		T2DM patients with depression Multiple race (36/38) (5.5%/31%)	UB, United States		C, 54.0 (8.4) I, 54.8 (8.8)	All women,84	6 months		Effectiveness of group CBT for depression and glucose control in women with T2DM	C, usual care I, group CBT	-0.4 mean change in HbA1c -13.8 mean change in fasting glucose -15.1 mean change in depression -10.1mean change in state anxiety	4, Moderate
Petrak et al. (40)		T1DM and T2DM patients with depression German (125/126) (18.4%/27%)	SB, Germany		C, 47.9(12.8) I, 49.0(10.6)	C, 48/77 I, 47/79	3 months		Assess the effectiveness of diabetes-specific CBT group therapy on depression and glucose in patients with diabetes	C, usual care plus sertraline I, same as control group plus group CBT	-0.25% mean change in HbA1c -12.64 mean change in depression	8, high	
Piette et al. (41)		T2DM patients with depression Multiple race(84% White) (167/172) (12.57%/15.7%)	UB, United States		C, 56.0 (10.9) I, 55.1 (9.4)	C, female (77.8%) I, female (64.9%)	12 months		Evaluate the impact of telephone-delivered CBT for depressive symptoms, physical activity, and diabetes-related outcome T2DM	C, usual care I, telephone-delivered CBT program	0.2 mean change in HbA1c -5.2 mean change in SBP -3.4 mean change in DBP -12.5 mean change in depression	7, Moderate	
Ridge et al. (42)		T1DM patients Multiple race (20% non-white) (117/106) (23.9%/30%)	UB, United Kingdom		C, 54.1 (10.4) I, 56.3 (10.3)	Full sample 36(28.1–43.8)	12 months		Assess the effect of MEF plus CBT on glycaemic control for T1DM	C, motivational enhancement therapy (four sessions) I, same as control group plus CBT	-0.578 mean change in HbA1c	5, Moderate
Safren et al. (43)		T2DM patients with depression Multiple race (42/45) (28.6%/20%)	SB, United States		C, 58.31 (7.41) I, 55.44 (8.72)	C, 22/20 I, 22/23	4 months		Assess the effectiveness of CBT for adherence and depression in patients with T2DM	C, usual care I, same as control group plus a CBT	-0.95 mean change in HbA1c -11.38 mean change in depression scale -1.98 mean change in CGI (clinical global impression)	8, High
Sharif et al. (44)		T2DM patients with depression Iranian (29/29) (3.3%/3.3%)	UB, Iran		C, 55 (10.5) I, 56 (11.2)	C, 3/26 I, 8/21	2 months		Evaluate the effectiveness of group CBT on depression and glucose control in T2DM	C, usual care I, group CBT	-1.09 mean change in HbA1c -5.81 mean change in depression	6, Moderate
Shayeghian et al. (45)		T2DM patients Iranian (53/53) (3.56%/1.18%)	UB, Iran		C, 55.70(8.98) I, 55.18 (8.26)	C, 26/27 I, 20/33	3 months		Evaluate the effectiveness of group-based ACT on self-management and glucose control in patients with T2DM	C, usual care (including education) I, same as control group plus group-based ACT (CBT approached based)	-0.36 mean change in HbA1c 6.98 mean change in Self-care 8.93-2 mean change in Acceptance and action diabetes	4, Moderate
Snoek et al. (46)		T1DM Dutch (41/45) (8%/27%)	UB, Netherlands		C, 37.4 (11.1) I, 38.1(9.7)	C, 14/27 I, 18/23	12 months		Effectiveness of group CBT in patients with T1DM	C, diabetes awareness education I, group CBT	No change in HbA1c -0.3 mean change in depression	5, Moderate
Sun et al. (47)		T2DM patients Chinese (45/45) (0%/0%)	UB, China		C,53.6 (9.7) I, 56.6 (9.7)	C, 27/18 I, 26/19	6 months		Evaluate the effectiveness of GCBT on health outcomes in T2DM	C, usual care I, same as control group plus group CBT	-0.24 mean change in HbA1c -1.48 mean change in 2hPG 1.41 mean change in FINS -3.53 mean change in anxiety -5.12 mean change in depression	**5,** **Moderate**
van Son et al. (48)		T1DM and T2DM with lower emotional wellbeing Dutch (69/70) (10%/18.84%)	UB, Netherlands		C, 57 (13) I, 56 (13)	C, 37/32 I, 33/37	3 months		Examine mindfulness-based cognitive therapy on emotion and glucose control of patients with diabetes	C, usual care I, CBT (MBCT)	-0.1 mean change in HbA1c -5.1mean change in Perceived stress -1.9 mean change in anxiety -2.6 mean change in depression	6, Moderate
Weinger et al. (49)		T1DM and T2DM patients Non-Hispanic white (75/74) (8%/4%)	UB, United States		C, 54.7 (25.0–75.1) I, 51.8 (23.7–74.2)	C, 39/36 I, 40/34	3 months		Assess the effect of structured behavioral intervention (based on CBT) for poorly controlled diabetes	C, attention control (focus on goal-setting) I, same as control group plus CBT-based structured behavioral intervention	-0.82 mean change in HbA1c -0.5 mean change in BMI -2.5 mean change in LDL-C -1.8 mean change in HDL-C 686 mean change in steps (pedometer readings)	7, Moderate
Welschen et al. (50)		T2DM patients Caucasian (78/76) (2.56/5.26%)	UB, Netherlands		C, 61.2 (8.8) I, 60.5 (9.4)	C, 50/28 I, 45/32	6 months		Examine the effects of CBT for reducing the risk of CHD in patients with T2DM	C, usual care I, same as control group plus CBT	-0.1 mean change in HbA1c -0.1 mean change in fasting blood glucose -0.3 mean change in TC 0.9 mean change in weight 0.2 mean change in BMI -1.2 mean change in SBP -2.4 mean change in DBP -1.2 mean change in depression	7, Moderate
Whitehead et al. (26)		T2DM patients NZ European And Maori (34/39) (26.5%/7.6%)	DB, Australia		C, 53.76 (8.68) I, 56.1 (6.91)	C, 20/14 I, 17/22	6 months		Examine whether a nurse-led education intervention alone of intervention using education and ACT was effective in reducing *HbA1c*	C, usual care (including education) I, same as control group plus ACT (CBT based)	-0.04 mean change in HbA1c -0.04 mean change in anxiety -0.54 mean change in depression	8, High
Wroe et al. (51)		T2DM patients with depression or anxiety NR (52/63) (0%/0%)	DB, United Kingdom		C, 63.63 (10.71) I, 63.48 (11.04)	C, 25/27 I, 28/35	6 weeks		Evaluate the effect of CBT-based psychological intervention focusing on depression and anxiety for T2DM	C, usual care I, CBT-based intervention	-0.03 mean change in HbA1c -3.4 mean change in depression -2.86 mean change in anxiety	7, Moderate

NR, not reported; CBT, cognitive behavioral therapy; C/I, control/intervention group; DO, drop out; UB, unblinded; DB, double blinded; SB, single blinded; T1DM, type 1 diabetes mellitus; T2DM, type 2 diabetes mellitus; TC, total cholesterol; HDL-C, high-density lipoprotein cholesterol; LDL-C, low-density lipoprotein cholesterol; TG, triglyceride; BMI, body mass index; SBP, systolic blood pressure; DBP, diastolic blood pressure; 2hPG, 2-h plasma glucose; FINS, fasting serum insulin; SANAD, Saudi Arabia networking aiding diabetes; MBCT, mindfulness-based cognitive therapy; ACT, acceptance and commitment.

All 23 studies reported data for some of the outcomes. Twenty-two studies provided full data of HbA1c. One study found no change in HbA1c after intervention and did not report the data of post-treatment HbA1c; this study was not included in the meta-analysis for HbA1c (46) but was included in the meta-analysis for depression symptoms.

### Post- to Pre-Treatment Effects of CBT-Based Intervention

#### Effects on Glycaemic Control

**Figure 2:**
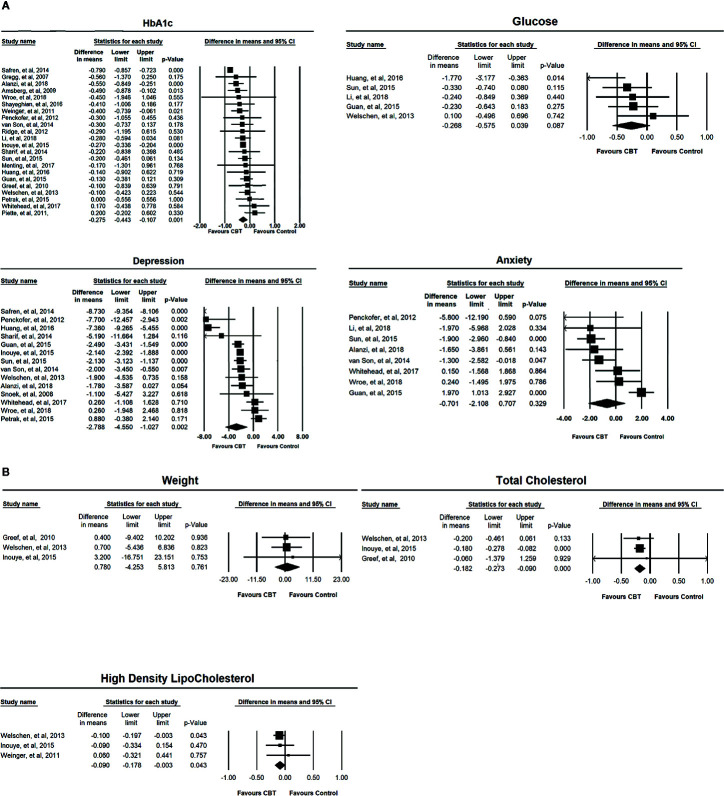
**(A)** Forest plots of the effects of CBT-based intervention on HbA1c, depressive symptom, anxiety symptom, and fasting blood glucose. **(B)** Forest plots of the effects of CBT-based intervention on weight, total cholesterol, and high-density lipoprotein cholesterol.

Twenty-two studies (n = 2619) were included in the random-effects meta-analysis comparing the effects on HbA1c of CBT-based interventions and the control condition. The results showed that CBT-based interventions were more effective than the control condition, with a mean reduction of 0.275% (95% CI: −0.443 to −0.107; *p < *0.01) and moderate effect size Hedge’s g 0.466 (95% CI 0.719 to 0.189, *p < *0.01). The heterogeneity of these studies was statistically significant (*I^2^ = *87.007%, *p < *0.001) (**Table 2**). Five studies with a total sample of 438 patients measured the level of FPG. As shown in **Table 2**, the meta-analysis found no significant reduction in FPG (−0.268, 95% CI: −0.575 to 0.039; *p > *0.05). The heterogeneity was not statistically different (*I^2^ = *32.915%, *p* > 0.05). The forest plots of the effect on HbA1c and FPG are presented in **Figure 2A**.

**Table 2:** Total effect of CBT on HbA1c, FPG, depressive symptom, anxiety symptom, weight, total cholesterol, and HDL-C.

Index	*Outcomes: post- to pre-treatment effect*					
	Studies, n	Participants	*I^2^ (%)*	Q-test	Mean difference (95% CI)	*P*
HbA1c	22	2619	87.007^***^	161.625	-0.275 (-0.443, -0.107)	0.001
FPG	5	438	32.915	5.963	-0.268 (-0.575, 0.039)	0.087
Depressive symptom	14	1866	97.230^***^	464.783	-2.788 (-4.450, -1.027)	0.002
Anxiety symptom	8	663	81.679^***^	38.207	-0.701 (-2.108, 0.707)	0.329
Weight	3	402	0.000	0.063	0.780 (-4.253, 5.813)	0.761
Total Cholesterol	3	402	0.000	0.053	-0.182 (-0.273, -0.090)	0.000
HDL-C	3	510	0.000	0.638	-0.090 (-0.178, -0.003)	0.043

***p < 0.001.

HbA1c, Haemoglobin A1c; FPG, fasting blood glucose; HDL-C, high-density lipoprotein cholesterol.

Subsequent subgroup analyses were performed to examine the effect on HbA1c of CBT-based interventions with different characteristics. As shown in **Table 3**, the subgroup analysis found that CBT-based interventions in the studies with the following characteristics had a better effect on HbA1c: low drop-out rate (less than 20%), CBT combined with other therapy, fewer sessions (<10), short treatment course (<6 weeks), use of a diabetes-specific manual, patients without mood symptoms, treatment delivered in groups and face-to-face. CBT-based interventions showed a significant effect on HbA1c in studies that included either T1DM or T2DM or both T1DM and T2DM, and the effect for T1DM was much better than the effect for T2DM and both T1DM and T2DM.

**Table 3:** Subgroup analysis on the effect of CBT on HbA1c.

Subgroups	*HbA1cs: post- to pre-treatment effect*												
	Studies(n)	Participants(n)	*I^2^ (%)*	Q-test	Mean difference(95% CI)	*P _between_*	Subgroups	Studies	Participants	I^2^ (%)	Q-test	Mean difference(95% CI)	P _between_
**Drop-out**						0.368	**Diabetic-specific manual**						0.888
<20%	19	2244	88.839	161.271	-0.260 (-0.442, -0.079)^**^		No	6	789	96.382	138.199	-0.311(-0.606, -0.015) ^*^	
≥20%	3	375	0.000	0.298	-0.430 (-0.752, -0.108)^*^		Yes	16	1830	0.000	14.406	-0.286(-0.413, 0.162)^***^	
**Intervention type**						0.821	**Treatment** **form**						0.394
Single CBT	12	1600	92.329	143.394	-0.295 (-0.524, -0.067)^*^		Individual	4	632	68.120	9.410	-0.300 (-0.488, -0.113)^*^	
Combined CBT	10	1019	26.198	12.195	-0.261 (-0.450, -0.073)^**^		Group	18	1987	88.285	145.108	-0.300 (-0.488, -0.013)^**^	
**Number of sessions**						0.939	**Treatment** **delivery way**						0.712
<10	11	1096	0.000	7.769	-0.276 (-0.336, -0.217)^***^		Face-to-face	18	2082	88.645	149.719	-0.290 (-0.475, -0.104) ^**^	
≥10	11	1523	86.067	71.773	-0.265 (-0.555, 0.026)		Remote	4	537	65.410	8.673	-0.200 (-0.637, 0.236)	
**Duration of session**						0.996	**Homework**						0.724
<90min	11	1350	87.302	78.750	-0.273 (-0.550, 0.004)		No	11	1094	85.661	69.740	-0.315 (-0.581, -0.048)^*^	
≥90min	11	1269	0.000	5.485	-0.274 (-0.334, -0.213)^***^		Yes	11	1525	0.000	8.508	-0.265 (-0.326, -0.205)^***^	
**Treatment course**						0.877	**Cognitive strategy**						0.485
<6 weeks	7	819	0.000	4.175	-0.266 (-0.328, -0.204)^***^		No	8	723	12.539	8.004	-0.344 (-0.508, -0.180)^***^	
≥6 weeks	15	1800	82.269	78.957	-0.285 (-0.512, -0.057)^*^		Yes	14	1896	91.386	150.919	-0.245 (-0.469, -0.021) ^*^	
**Combined with mood symptoms**						0.880	**Behavioral experiment**						0.985
No	13	1545	0.000	10.374	-0.274(-0.332, -0.217) ^***^		No	19	2223	82.287	101.621	-0.269 (-0.464, -0.074) ^**^	
Yes	9	1074	85.465	55.038	-0.247(-0.587, 0.092)		Yes	3	396	0.000	0.061	-0.271 (0.001, -0.336)^***^	
**Type of diabetes**						0.951	**Stress management**						
Type 1	6	596	0.000	2.565	-0.242 (-0.407, -0.076)**		No	12	1419	83.619	67.153	-0.328(-0.577, -0.079) ^*^	0.612
Type 2	12	696	92.526	147.174	-0.283 (-0.522, -0.044) *		Yes	10	1200	2.065	9.190	-0.260 (-0.334, -0.187)^***^	
Type 1 and 2	4	1327	0.000	1.452	-0.295 (-0.524, -0.065) *		**Mood management**						0.438
**Interpersonal strategies**						0.754	No	7	741	0.000	4.836	-0.353 (-0.524, -0.183)^***^	
No	15	2146	91.103	157.353	-0.275 (-0.479, -0.071) ^*^		Yes	15	1878	90.963	154.912	-0.247 (-0.454, -0.041)^*^	
Yes	7	473	0.000	1.503	-0.304 (-0.502, -0.106) ^**^								

*p < 0.05; **p < 0.01; ***p < 0.001.

The subgroup analyses also examined the effects on HbA1c of CBT-based interventions with different technique components. CBT-based interventions showed a better effect on HbA1c when they used the following components: behavioral experiment, stress management, homework assignment and interpersonal strategy. In addition, CBT-based interventions that did not use cognitive strategies and mood management were more effective than CBT-based interventions that used cognitive strategies and mood management (**Table 3**).

#### Effects on Depression and Anxiety Symptoms

The effect on depression symptoms was analysed in 14 studies that had a total sample of 1,866 participants. As shown in **Table 2**, CBT-based interventions were more beneficial for depression symptoms than for the control condition with mean reduction of depression of −2.788 (95% CI: −4.450 to −1.027; *p* < 0.01), and large effect size of Hedge g 0.966 (95% CI 1.507 to 0.426, *p* < 0.001). There was significant heterogeneity between these studies (*I^2^* = 97.230%, *p* < 0.001). Eight studies with a total sample of 663 participants were included in the random-effects meta-analysis for anxiety symptoms, and the result showed no significant difference between CBT-based interventions and control conditions (−0.701, 95% CI: −2.108 to 0.707; *p* > 0.05). The heterogeneity for these studies was statistically significant (*I^2^ = *81.679%, *p < *0.001) (**Table 2**). The forest plots of the effect on mood symptoms are presented in **Figure 2A**.

Subgroup analyses were performed to examine the effect of CBT-based interventions with different characteristics on improving depression symptoms. As shown in **Table 4**, the results demonstrate that CBT-based interventions with the following characteristics had a better effect on depression symptoms: single CBT, longer treatment course (>6 weeks), shorter duration of session (< 90 min) and no use of diabetes-specific manual. CBT-based interventions had a significant effect for patients with mood symptoms or without mood symptoms, and the effect was much better for patients without mood symptoms than it was for those with mood symptoms.

**Table 4:** Subgroup analysis on the effect of CBT on depressive symptoms.

Subgroups	*Depressive symptom: post- to pre-treatment effect*												
	Studies(n)	Participants(n)	*I^2^ (%)*	Q-test	Mean difference(95% CI)	*P _between_*	Subgroups	Studies	Participants	I^2^ (%)	Q-test	Mean difference(95% CI)	P _between_
**Intervention type**						0.926	**Diabetic-specific manual**						0.609
Single CBT	10	1288	97.682	421.048	-2.849 (-5.090, -0.608) ^*^		No	6	789	98.677	375.113	-3.274(-5.964, -0.583) ^*^	
Combined CBT	4	389	92.654	40.838	-2.673 (-5.641, 0.295)		Yes	8	888	89.339	65.662	-2.338(-4.714, 0.038)	
**Duration of session**						0.064	**Homework**						0.739
<90min	7	1512	97.189	21.3437	-4.197 (-7.144, -1.250) ^**^		No	7	632	97.940	291.233	-2.401(-5.535, 0.733)	
≥90min	7	965	85.432	41.187	-1.102 (-2.522, 0.318)		Yes	7	1045	89.516	57.231	-3.206(-4.951, -1.101) ^**^	
**Treatment course**						0.065	**Cognitive strategy**						0.935
<6 weeks	5	635	74.702	15.812	-0.964 (-2.422, 0.494)		No	4	308	92.653	40.835	-2.677(-6.096, 0.741)	
≥6 weeks	9	1042	97.457	314.630	-3.897(-6.653, -1.140) ^**^		Yes	10	1369	97.866	421.836	-2.845(-5.026, -0.664) ^*^	
**Combined with mood symptom**						0.214	**Behavioral experiment**						0.600
No	6	783	60.010	12.503	-1.699(-2.516, -0.881) ^***^		No	10	1195	97.527	363.928	-2.968(-5.561, -0.374) ^*^	
Yes	8	894	97.644	297.134	-3.861(-7.173, -0.549) ^*^		Yes	4	482	69.957	9.986	-2.058(-4.255, 0.138)	
**Stress management**						0.526	**Mood management**						0.094
No	8	1118	97.862	327.356	-2.272 (-5.165, 0.621)		No	3	289	14.644	2.343	-1.156(-2.506, 0.194)	
Yes	6	559	89.347	46.934	-3.479(-5.839,-1.120)^**^		Yes	11	1388	97.805	455.614	-3.258(-5.314, -1.20) ^**^	
**Interpersonal strategies**						0.059							
No	10	1398	97.941	437.142	-2.022 (-4.014, -0.030) ^*^								
Yes	4	279	58.092	7.158	-5.592 (-8.712, -2.471)^***^								

*p < 0.05; **p < 0.01; ***p < 0.001.

The subgroup analyses also examined the effects of CBT-based interventions with different technique components on improving depression symptoms. CBT-based interventions showed a better effect when they used the following components: cognitive strategy, homework assignment, stress management, mood management, and interpersonal strategy. In addition, CBT-based interventions that did not use behavioral experiment showed a better effect on the reduction of depression symptom than those that did use behavioral experiment (**Table 4**).

#### Effects on Weight, Total Cholesterol, and HDL-C

Three studies with a total sample of 402 patients measured weight change. As shown in **Table 2**, there was no difference in weight change between CBT-based intervention and control condition (0.780, 95% CI: −4.253 to 5.813; *p* > 0.05). The heterogeneity for these studies was not significant (*I^2^* = 0%, *p* > 0.05).

Three studies (n = 402) measured total cholesterol before and after treatment. CBT-based intervention significantly reduced the total cholesterol, with a reduced pooled mean across these studies of −0.182 mmol/L (95% CI: −0.273, −0.090; *p < *0.001) with small effect size of Hedge’s g of 0.185 (-0.377 to 0.007, P=0.06). There was no significant difference in the heterogeneity (*I^2^* = 0%, *p* > 0.05) (**Table 2**).

Three studies (n = 510) included measurement of HDL-C before and after intervention. The meta-analysis found a significant difference in HDL-C change between CBT-based interventions and control condition (−0.090, 95% CI: −0.178 to −0.003; *p < *0.05). The heterogeneity was not statistically significant for these studies (*I^2^* = 0%, *p* > 0.05) (**Table 2**).

The forest plots of the effect on weight, total cholesterol and HDL are presented in **Figure 2B**.

### Publication Bias

The one-study-removed methods were used to assess sensitivity, and it was found that removing one study at a time did not change the overall results for all outcome variables. Cumulative analysis also indicates with only three studies the overall results are significant. As shown in **Table 5**, the Egger test suggested a minimal publication bias for all variables. Funnel plots of all outcome variables are presented in **Supplementary Figures 1** and **2**.

**Table 5:** Egger’s regression analysis on publication bias of included studies.

Variables	T value	95% CI	P-value
HbA1c	1.698	-0.289, 2.828	0.105
FPG	1.314	-6.710, 2.788	0.280
Depressive symptom	0.159	-5.341, 4.611	0.876
Anxiety symptom	1.081	-6.534, 2.529	0.321
Weight	1.245	-2.586, 3.148	0.431
Total cholesterol	0.923	-10.010, 15.479	0.453
HDL-C	1.462	-5.777, 7.279	0.382

HbA1c, Haemoglobin A1c; FPG, fasting plasma glucose; HDL-C, high-density lipoprotein cholesterol.

## Discussion

This expanded meta-analysis aimed to evaluate whether CBT-based interventions have a greater effect on glycaemic control, mood symptoms, and physiological outcomes for adult patients with T1DM or T2DM compared to usual care and non-CBT interventions. The results showed that CBT-based interventions had a better effect on reducing HbA1c levels, depression symptoms and total cholesterol compared with the control condition. We also did the subgroup analyses and found several key factors that influence the effect of CBT.

### Effect on Glycaemic Control

In congruence with previous meta-analyses, we found that CBT-based interventions significantly reduced HbA1c levels in patients with either T1DM or T2DM (5, 12). We found that CBT-based interventions have moderate effect size on glycaemic control. The concentration of HbA1c represents the average level of blood sugar for nearly 2–3 months. It is reported that a lower HbA1c level is helpful for reducing the risk of DM-related complications. However, many patients with DM have inadequate glycaemic control arising from inadequate self-care, low adherence to treatment, and comorbid physical or psychological complications (2). According to the theory of CBT, behavioral and emotional problems are maintained by dysfunctional automatic thoughts and schemas. Thus, restructuring cognitions using cognitive and behavioral techniques can reduce dysfunctional behaviors (16). The findings of the current meta-analysis further support the benefits of applying CBT-based interventions in the management of DM. Our results are different from previous meta-analysis which showed CBT had small effect on HbA1c, that CBT-based interventions have moderate effect size on glycaemic control (7). It is possible we have bigger number of studies than previous studies which may have generate more power than the published study (7).

Patient adherence to therapy has a great influence on the therapeutic effect (52–54). In this meta-analysis, most CBT-based interventions had a low drop-out rate, and the subgroup analyses showed that the interventions with a low drop-out rate had a better effect than those with a higher drop-out rate. This finding demonstrates that CBT is easily accepted by patients with DM, and better compliance is helpful for glycaemic control. We also found that CBT-based interventions with fewer sessions (<10) and a shorter treatment course (<6 weeks) had a better effect on glycaemic control. This result may be related to the treatment compliance. We speculate that the shorter duration of treatment is easier for patients to accept, while long-term treatment will lead to a decrease in patients’ treatment compliance for some reasons (e.g., cost of time and physical discomfort), thus affecting the effect of treatment. This finding suggests that the duration of CBT-based interventions is not characterized by ‘the longer the better’; that is, a brief CBT intervention may be a better choice for community patients with DM.

Using a targeted manual is an important basis for CBT (9). Most studies included in our meta-analysis developed a diabetes-specific CBT manual, and showed a better treatment effect than the studies that did not use a diabetic-specific manual. We found the studies using CBT combined with other therapies had a better effect on glycaemic control than studies that used CBT alone. The current findings demonstrate that activating patients’ motivation, delivering diabetic education and practising physical activity may enhance the effect of CBT. Following the ADA’s argument that comprehensive therapy may have more advantages for improving glycaemic control (1), it is of great practical value for clinical management to integrate more useful methods within a CBT framework and to compile a detailed diabetes-specific manual.

We found that intervention delivered in group form had a better effect on glycaemic control than intervention delivered in individual form. Group CBT, which emphasizes the influence of social factors on individual behavior, has been found to have more advantages for some diseases because participants can access more opportunities for positive peer modeling and the normalization of their symptoms through communicating with group members (55). Remotely delivered CBT (e.g., *via* mobile phone and internet) has been developed and found by several studies to be effective for glycaemic control (17–20). However, our subgroup analysis found that face-to-face delivered CBT was much better than remotely delivered CBT. This may be because most face-to-face therapies were organized in groups form, and this kind of treatment structure is more effective.

We also explored which types of patients benefit more from CBT-based interventions. We found CBT-based interventions were effective for patients with T1DM and for those with T2DM, suggesting that CBT can be widely used in the treatment of various types of DM. Further, the subgroup analysis indicated that CBT-based interventions had a better effect for patients with T1DM. Patients with T1DM need lifelong insulin therapy and are at high risk of various complications. Therefore, they may be facing more disease-related stress, which is an important target of CBT-based intervention. CBT that is specifically focused on diabetes may guide patients to accept and understand the disease, as well as to practice effective behavior skills to better control blood sugar. Patients with DM have a high risk of also having depression and anxiety symptoms, which can have a negative effect on the treatment of DM (4). Not surprisingly, we found the patients with DM combined with mood symptoms benefited less from the intervention for improving glycaemic control. Thus, it is important to evaluate the mood symptoms for patients with DM, and offer effective treatment for mood during the DM management.

The subgroup analyses also examined the effect on HbA1c of CBT-based interventions that used different technique components. CBT-based interventions showed a better effect on HbA1c when they used the following components: behavioral experiment, stress management, homework assignment and interpersonal strategy. These components are key techniques of CBT. This finding is consistent with the findings of previous research demonstrating CBT should emphasize these components (25–27). Further, CBT-based interventions without using cognitive strategies and mood management were more effective than those using cognitive strategies and mood management. As stated, shorter-term interventions had a better effect on glycaemic control. However, these short-term interventions may hinder the performance of cognitive strategies and mood management because such treatments usually require longer periods to be conducted systematically and deeply. Thus, cognitive strategy and mood management did not show a better effect on glycaemic control, although they are the core components of CBT. An additional reason for this result is that patients with DM may be principally concerned about their glycaemic control, rather than about their cognitive and emotional changes. According to the current findings, we speculate that targeted behavioral interventions, including behavioral experiments, homework assignments and stress management, are more effective than cognitive strategies for glycaemic control. During a short treatment course, repeated behavioral practice helps individuals to develop effective self-care behaviors for DM, and thus results in better glycaemic control.

We analyzed change of FPG before and after intervention. Unlike another meta-analysis (5), the current meta-analysis found that the effect of CBT-based interventions on FPG was not statistically significant. FPG is a commonly used clinical indicator of blood sugar level, but recent studies have found that it has a high rate of variation and low repeatability (56). The 24 trials included in this meta-analysis observed the change of HbA1c before and after interventions, while only five of these observed the change of FPG. Thus, the small sample size of studies measuring FPG and the varying nature of FPG may explain why this meta-analysis found no effect on FPG.

### Effect on Mood Symptoms

CBT has been found to be effective in treating adult depression and depression symptoms related to other conditions such as stroke and chronic pain (57, 58). Uchendu and Blake (12) performed a review and meta-analysis on the effect of CBT on psychological outcomes in adults with DM, including only nine RCTs, and found CBT could improve short-, medium-and long-term depression. In this study, we included 14 RCTs measuring depression symptoms, and found that CBT-based interventions had a larger effect on these symptoms. Our expanded study provides further evidence for the effect of CBT on depression in adult patients with DM.

The subgroup analyses showed CBT-based interventions with the following characteristics had a better effect on reducing depression symptoms: patients without mood symptoms, homework assignment, stress management, and interpersonal strategy. These findings are consistent with the results of subgroup analyses of the effect for HbA1c. Further, we found several intervention strategies for improving depression symptoms that differed with that for glycaemic control. The results showed that interventions using single CBT, with a longer treatment course (>6 weeks), and using a cognitive strategy and mood management, had a better effect on improving depression symptoms. Depressed mood is related to uncontrolled negative thoughts and dysfunctional behavior (8). Thus, the improvement of depression symptoms may be attributed to the alteration of dysfunctional cognition induced by CBT-based intervention. As explained, the progress of cognitive reconstruction requires a longer treatment course. In addition, the intervention that did not use a diabetes-specific manual had a better effect on depression symptoms. It seems logical that treatment using a diabetes-specific manual focused on the management of glycaemic control had less effect on depression symptoms. The current findings suggest that traditional CBT with generic components has greater benefit in improving depression symptoms. Thus, in clinical practice, it is necessary to compile specific manuals with different technical components according to the population and purpose of treatment.

CBT has been found to have a positive effect on anxiety (59). Chapman and colleagues reviewed the effect of psychological interventions on psychological outcomes for patients with T2DM in China (5). They found CBT was more effective in the reduction of depression and anxiety compared with control condition. Uchendu and Blake also found that CBT could improve short- and medium-term anxiety in patients with DM (12). However, we did not find any effect of CBT on anxiety in this study. Only five studies using anxiety as an outcome were included in this meta-analysis, and the anxiety symptoms reported were relatively mild. Thus, our findings should be interpreted with caution. Anxiety is common in patients with DM, and has a bad influence on the management of DM. More research is needed to prove the effect of CBT on the anxiety related to DM.

### Effect of CBT on DM-Related Physiological Outcomes

We evaluated the effect of CBT-based interventions on weight, total cholesterol, and HDL-C. CBT-based interventions show small effect on reducing total cholesterol than control condition. However, there was also a slight decrease of HDL-C after intervention in trials with CBT-based intervention. No statistically significant effect on weight was found in this study. There is interaction between lipoprotein concentration, glucose concentration, and weight. Thus, effective glycaemic control is helpful for the management of blood lipids and weight (60). Our current results should be explained cautiously because the physiological indicators are affected by various factors during the progress of DM. In addition, small number of trials with these indexes were included in this meta-analysis. More research is needed to explore whether CBT-based interventions can improve different physiological indicators related to DM.

## Limitations

This study included 23 RCTs using CBT-based interventions as treatment for DM, and yielded high-quality evidence. This expanded meta-analysis covered various CBT-based interventions for patients with either T1DM or T2DM, including single CBT and combined CBT, traditional CBT and newly developed CBT (MBCT and ACT), CBT delivered face-to-face and CBT delivered remotely. We aimed to perform a comprehensive meta-analysis of the value of CBT in the treatment of DM, but our research has some limitations that must be acknowledged. First, we found high heterogeneity in this meta-analysis, although we conducted various subgroup analyses. The heterogeneity may be caused by a combination of some confounding factors, including clinical and methodological diversity. Second, we examined only the effect of intervention at the post-intervention, and did not include follow-up findings, thus we gathered no evidence on whether CBT-based interventions have a long-term effect on glycaemic control and depression symptoms. Third, although most studies included in this meta-analysis used a CBT manual, the concrete setting and contents of the manuals varied among the different studies. Regular acceptance of the supervision of experienced psychotherapists is a necessary measure to ensure the quality of CBT. However, few studies mentioned supervision during the CBT intervention. Lastly, the absence of treatment integrity within the included studies is also an important limitation. Thus, the quality of CBT implemented in the studies is uncertain. Given these limitations, the results of this meta-analysis should be interpreted with caution.

## Conclusion

Our findings further demonstrate that CBT-based interventions are effective in improving glycaemic control and depression symptoms for patients with either T1DM or T2DM. In addition, several mediators of the effect of CBT were found through subgroup analyses. The interventions that emphasized homework assignments, stress management, and interpersonal strategy, and that were delivered *via* group had a larger effect on both HbA1c and depression symptoms. However, implementing a behavioral strategy showed a better effect for glycaemic control, and implementing a cognitive strategy showed a better effect for depression symptoms. The current results suggest that it is necessary to adopt technical components of CBT including cognitive and behavioral components to improve clinical outcomes and psychological wellbeing in patients with diabetes. Given the overall results of this review, we recommend the provision of CBT-based interventions for improving the management of DM, which may ultimately enhance glycaemic control and improve psychological wellbeing of the patients.

## Data Availability Statement

All datasets generated for this study are included in the article/**Supplementary Material**.

## Author Contributions

XY drafted and revised the manuscript. ZL reviewed the manuscript. JS designed the study, analyzed the results and plots, drafted manuscript, edited, and critically reviewed and revised the manuscript.

## Funding

1) Beijing Hospitals Authority Young Talents Training Program, China (QML20181903), and 2) Capital applied research on clinical characteristics of Beijing Science and Technology Commission, China (Z181100001718077).

## Conflict of Interest

The authors declare that the research was conducted in the absence of any commercial or financial relationships that could be construed as a potential conflict of interest.
