# Supplementary material for: Effects of Cognitive Behavioral Therapy–Based Intervention on Improving Glycaemic, Psychological, and Physiological Outcomes in Adult Patients With Diabetes Mellitus: A Meta-Analysis of Randomized Controlled Trials
Source: Front Psychiatry. 2020 Jul 28;11:711. doi: 10.3389/fpsyt.2020.00711 (PMC7399630; doi:10.3389/fpsyt.2020.00711)
Supplement: Supplementary file 1 [file DataSheet_1.docx]

Supplementary Table 1 Literature search strategy in different databases

| **Database Literature search strategy** | |
| --- | --- |
| Pubmed | Diabetes mellitus [mh] OR diabetes OR type 1 diabetes OR type 2 diabetes |
|  | Cognitive [mh] OR Cognitive behavio* therapy OR therapy, Cognitive behavio* OR Behavio* therapies, Cognitive OR Behavio* therapy, Cognitive OR Cognitive behavio* therapies OR Therapies, Cognitive behavio* OR Therapy, Cognitive Behavio* OR CBT |
|  | Mindfulness-based cognitive therapy [mh] OR Mindfulness OR MBCT |
|  | Accept and commitment [mh] OR ACT |
|  | Randomized controlled trials as Topic [mh] OR Clinical trials, randomized) OR Trials, Randomized clinical OR Controlled clinical Trials, Randomized OR Randomized controlled trial [Publication type] |
| Cochrane Library | “diabetes mellitus”: ti, ab, kw OR “diabetes”: ti, ab, kw |
|  | MeSH description: [Cognitive therapy] explode all trees OR Cognitive therapy: ti, ab, kw OR Cognition therapy: ti, ab, kw OR Cognition therapies: ti, ab, kw OR Therapies, Cognition: ti, ab, kw OR Cognitive behavior therapies: ti, ab, kw OR Therapies, Cognitive behavior: ti, ab, kw OR Cognitive behavioral therapy: ti, ab, kw OR Behavioral therapies, Cognitive: ti, ab,kw OR Cognitive behavioral treatment: ti, ab, kw OR CBT: ti, ab,kw  MeSH description: [Mindfulness-based cognitive therapy] explode all trees OR Mindfulness: ti, ab,kw OR MBCT: ti, ab, kw |
|  | MeSH description: [Accept and commitment] explode all trees: ti, ab, kw OR ACT: ti, ab, kw |
|  | MeSH description: [Randomized controlled trials] explode all trees OR Randomized controlled trial: it, ab, kw OR Randomized controlled clinical trial: ti, ab, kw OR randomized controlled study：ti, ab, kw OR Randomized controlled studies: ti, ab, kw  (All word variations have been searched) |
| Scopus | Full text: diabetes mellitus OR Full text:diabetes |
|  | Full text: Cognitive therapy OR Full text: Cognitive behavio* therapy OR Full text: Behavio* therapy OR Full text: Cognitive behavior treatment |
|  | Full text: Mindfulness-based cognitive therapy OR Full text: MCBT |
|  | Full text: Accept and commitment OR Full text: ACT |
|  | Full text: randomized controlled trials OR randomized controlled study |
| Embase | ‘diabetes mellitus’ / exp OR diabetes |
|  | ‘cognitive behavioral therapy’/ exp OR ‘cognitive therapy’/exp OR ‘cognitive therapies’ OR ‘therapies, cognitive’ OR ‘cognition therapy’ OR ‘Cognition therapies” OR ‘behavior therapy’ OR ‘Cognitive behavior therapy’ OR ‘Cognitive behavior therapies’ OR ‘behavioral therapies, cognitive’ OR ‘behavioral therapy, cognitive’ OR ‘cognitive behavioral treatment’ |
|  | ‘Mindfulness-based cognitive therapy’/ exp OR ‘mindfulness’ OR ‘MBCT’ |
|  | ‘Accept and commitment’ /exp OR ‘ACT’ |
|  | ‘randomized controlled trial’/exp OR ‘randomized controlled trial (topic)’/exp OR random* |
| ProQuest Dissertations and Theses | Diabetes mellitus: ti, ab, OR Diabetes: ti, ab  Cognitive therapy: ti, ab OR Cognition therapy: ti, ab OR Cognition therapies: ti, ab OR Therapies, Cognition: ti, ab OR Cognitive behavior therapies: ti, ab OR Therapies, Cognitive behavior: ti, ab OR Cognitive behavioral therapy: ti, ab OR Behavioral therapies, Cognitive: ti, ab OR Cognitive behavioral treatment: ti, ab OR CBT: ti, ab  Mindfulness-based cognitive therapy: ti, ab OR Mindfulness: ti, ab OR MBCT: ti, ab |
|  | Accept and commitment: ti, ab OR ACT: ti, ab |
|  | Randomized controlled trial: it, ab OR Randomized controlled clinical trial: ti, ab OR randomized controlled study: ti, ab OR Randomized controlled studies: ti, ab |

Supplementary Table 2 Overview of settings and components of CBT-based interventions of included studies

| Study (year) | Treatment  form | Intervention  type | Delivery way | Diabetic-specific manual | Number of sessions | Duration of session | Treatment course | Cognitive strategy | | Behavioural experiment | | Mood management | | Stress manage-ment | Homework  assignment | Interpersonal strategy |  |
| --- | --- | --- | --- | --- | --- | --- | --- | --- | --- | --- | --- | --- | --- | --- | --- | --- | --- |
| Alanzi et al. (2018) | 0 | 1 | 1 | 1 | 0 | 0 | 1 | 0 | 0 | | 0 | | 0 | | 0 | **0** | |
| Amsberg et al. (2009) | 1 | 0 | 0 | 1 | 1 | 1 | 1 | 0 | 0 | | 1 | | 1 | | 1 | **1** | |
| Greef et al. (2010) | 1 | 1 | 0 | 1 | 0 | 1 | 1 | 1 | 0 | | 0 | | 0 | | 1 | 1 | |
| Guan et al. (2015) | 1 | 0 | 0 | 0 | 1 | 0 | 1 | 1 | 0 | | 1 | | 0 | | 0 | 0 | |
| Gregg, et al. (2007) | 1 | 1 | 0 | 1 | 0 | 1 | 0 | 0 | 0 | | 0 | | 0 | | 0 | 0 | |
| Huang et al. (2016) | 1 | 1 | 0 | 1 | 1 | 0 | 1 | 0 | 0 | | 1 | | 1 | | 1 | 1 | |
| Inouye et al. (2015) | 1 | 0 | 0 | 0 | 0 | 1 | 0 | 1 | 1 | | 1 | | 1 | | 1 | 0 | |
| Li et al. (2018) | 1 | 0 | 0 | 1 | 0 | 1 | 0 | 1 | 0 | | 1 | | 1 | | 0 | 1 | |
| Menting et al. (2017) | 0 | 0 | 1 | 1 | 1 | 0 | 1 | 1 | 0 | | 1 | | 1 | | 0 | 1 | |
| Penckofer et al. (2012) | 1 | 0 | 0 | 1 | 1 | 1 | 1 | 1 | 1 | | 1 | | 1 | | 1 | 1 | |
| Petrak et al. (2015) | 1 | 0 | 0 | 1 | 1 | 1 | 1 | 1 | 0 | | 1 | | 0 | | 1 | 0 | |
| Piette et al. (2011) | 0 | 1 | 1 | 1 | 1 | 0 | 1 | 1 | 0 | | 1 | | 1 | | 1 | 0 | |
| Ridge et al. (2012) | 1 | 1 | 0 | 1 | 1 | 0 | 1 | 1 | 0 | | 0 | | 0 | | 0 | 0 | |
| Safren et al. (2014) | 1 | 0 | 0 | 0 | 1 | 0 | 1 | 1 | 0 | | 1 | | 0 | | 0 | 0 | |
| Sharif et al. (2014) | 1 | 0 | 1 | 1 | 0 | 0 | 1 | 1 | 0 | | 1 | | 1 | | 1 | 1 | |
| Shayeghian et al. (2016) | 1 | 1 | 0 | 1 | 1 | 0 | 1 | 0 | 0 | | 1 | | 0 | | 1 | 0 | |
| Snoek et al. (2008) | 1 | 0 | 0 | 1 | 0 | 1 | 0 | 1 | 1 | | 1 | | 1 | | 1 | 1 | |
| Sun et al. (2015) | 1 | 0 | 0 | 0 | 1 | 0 | 1 | 1 | 0 | | 1 | | 0 | | 0 | 0 | |
| van Son et al. (2014) | 1 | 1 | 0 | 0 | 0 | 1 | 1 | 1 | 0 | | 1 | | 0 | | 1 | 0 | |
| Weinger et al.  (2011) | 1 | 1 | 0 | 1 | 0 | 1 | 0 | 0 | 0 | | 0 | | 1 | | 1 | 0 | |
| Welschen et al. (2013) | 0 | 0 | 0 | 0 | 0 | 0 | 0 | 0 | 0 | | 0 | | 0 | | 0 | 0 | |
| Whitehead et al. (2017) | 1 | 1 | 0 | 1 | 0 | 1 | 0 | 0 | 0 | | 1 | | 1 | | 0 | 0 | |
| Wroe et al. (2018) | 1 | 0 | 0 | 1 | 0 | 1 | 0 | 1 | 1 | | 0 | | 0 | | 0 | 0 | |

Treatment form: 0 = individual; 1 = group. Intervention type: 0 = single CBT; 1 = CBT combined with other methods;

Delivery way: 0 = face-to-face; 1 = remote (include telephone delivered or web delivered).

Diabetes-specific manual: specific manual combined CBT and diabetes mellitus; 0 = No; 1 = Yes.

Number of sessions: 0 = < 10; 1 = ≥10. Duration of session: 0 = < 90 min; 1 = ≥ 90 min.

Treatment course: 0 = < 6weeks; 1 = ≥ 6weeks.

Cognitive strategy: helps the individual to discover, challenge, and modify or replace their negative, irrational thoughts; 0 = No （not mentioned in the manual）; 1 = Yes (explicitly mentioned in the manual).

Behavioural experiment: planned activities to test the validity of beliefs and assumptions; 0 = No （not mentioned in the manual）; 1 = Yes (explicitly mentioned in the manual).

Mood management: identifying negative emotions, controlling and expressing emotions; 0 = No （not mentioned in the manual）; 1 = Yes (explicitly mentioned in the manual).

Stress management: help individual deal with stress by analysing the specific stressors and taking positive actions to decrease their effects; 0 = No （not mentioned in the manual）; 1 = Yes (explicitly mentioned in the manual).

Homework assignment: assign homework to individual as part of the treatment; 0 = No （not mentioned in the manual）; 1 = Yes (explicitly mentioned in the manual).

Interpersonal strategies: increasing an individual’s maladaptive behavioural patterns in relating to other people; 0 = No （not mentioned in the manual）; 1 = Yes (explicitly mentioned in the manual).


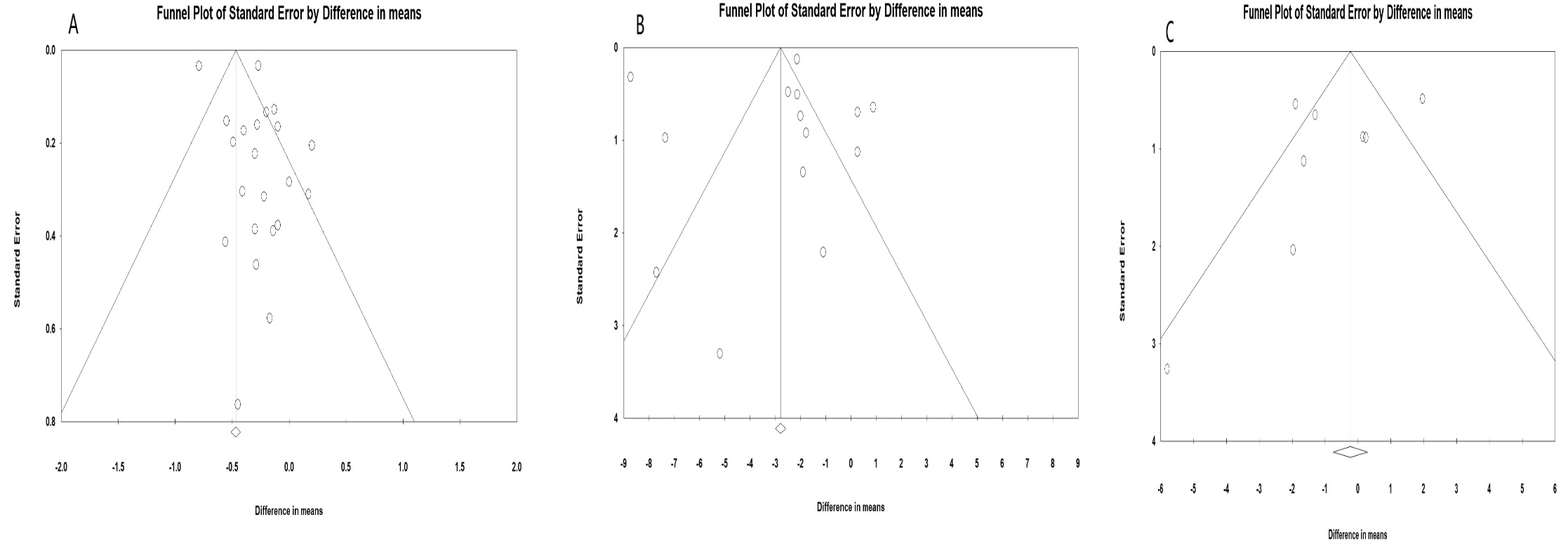


Supplementary Figure 1. Funnel plots of publication bias on HbA1c (A), depressive symptom (B), anxiety symptom (C).


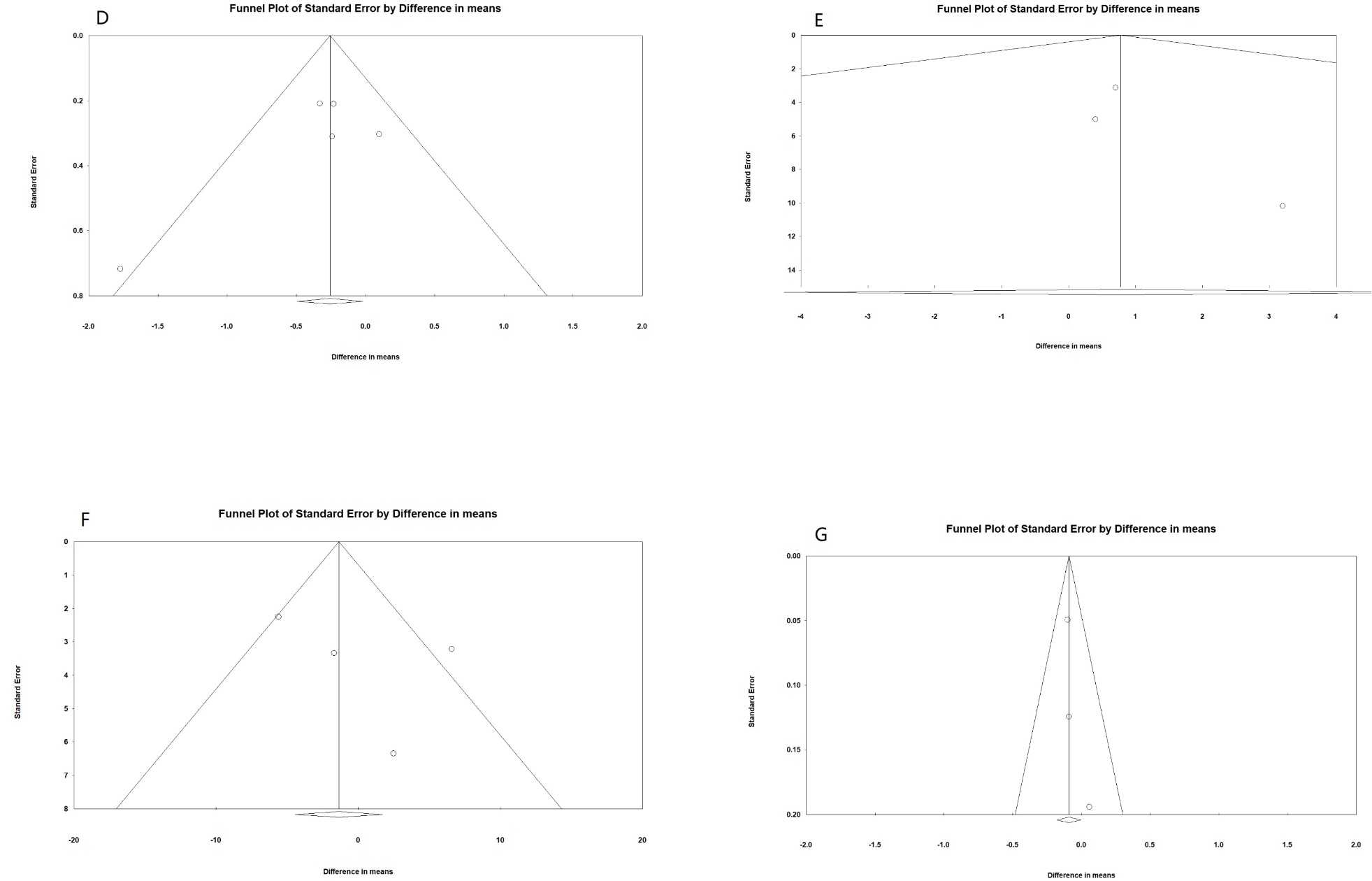


Supplementary Figure 2. Funnel plots of publication bias on fasting blood glucose (D), weight (E), total cholesterol (F) and high-density lipoprotein cholesterol (G).
